# Associations of Partnership Types and Quality on Cognitive Performance Among Midlife and Older Sexual Minority Men With or Without HIV

**DOI:** 10.1007/s10461-024-04501-0

**Published:** 2024-09-16

**Authors:** Moka Yoo-Jeong, Andrea M. Weinstein, Deanna Ware, Mark Brennan-Ing, Steven Shoptaw, Linda A. Teplin, Sabina A. Haberlen, M. Reuel Friedman, Michael W. Plankey

**Affiliations:** 1 School of Nursing, Bouvé College of Health Sciences, Northeastern University, Boston, MA USA; 2 Department of Psychiatry, University of Pittsburgh, Pittsburgh, PA USA; 3 Division of Internal Medicine, Department of Medicine, Georgetown University Medical Center, Washington, DC USA; 4 Brookdale Center for Healthy Aging at Hunter College, City University of New York, New York, NY USA; 5 Departments of Family Medicine and Psychiatry and Biobehavioral Sciences, University of California, Los Angeles, CA USA; 6 Department of Psychiatry and Behavioral Sciences, Northwestern University Feinberg School of Medicine, Chicago, IL USA; 7 Department of Epidemiology, Johns Hopkins University Bloomberg School of Public Health, Baltimore, MD USA; 8 Department of Urban-Global Public Health, School of Public Health, Rutgers University, Newark, NJ USA

**Keywords:** Partnership status, Partnership quality, Cognitive performance, Sexual minority men, Midlife, Older adults, HIV

## Abstract

Partnership status among sexual minority men (SMM) is a potentially important yet underexplored predictor of cognitive functioning. Using data from the understanding patterns of healthy aging among men who have sex with men substudy of the Multicenter AIDS Cohort Study, we assessed the associations of partnership status and quality with cognitive performance in middle-aged and older SMM, adjusting for sociodemographic and clinical covariates. Partnership status was classified into four types: “only a primary partnership,” “only a secondary partnership,” “both a primary and secondary relationship,” and “neither a primary nor secondary relationship.” Partnership quality was assessed based on perceived support or strain from partners. Cognitive performance was evaluated using the *z*-scores on the Symbol Digit Modalities Test (SDMT), Trail Making Test Parts A (TMT-A) and B (TMT-B), and a composite *Z*-score that summed the SDMT, TMT-A, and TMT-B *z*-scores. Among 1067 participants (median age 60, 85.7% college educated), having a primary partner was associated with better cognitive performance (*Z*-score composite $\widehat{\upbeta }$= 0.41 [95% CI 0.12–0.70]), TMT-A ($\widehat{\upbeta }$= 0.16 [95% CI 0.02–0.30]), and TMT-B ($\widehat{\upbeta }$= 0.19 [95% CI 0.06–0.33]). Support from secondary partners was also linked to better cognition. Additionally, there was a significant interaction between partnership and HIV status, indicating that SMM with HIV and both primary and secondary partners showed better cognitive outcomes than unpartnered SMM with HIV. These findings suggest that having a primary partner and receiving support from secondary partners may contribute to better cognitive health among middle-aged and older SMM, especially those with HIV.

## Introduction

Advancement of and access to combination antiretroviral therapy (cART) have increased the longevity of persons with HIV (PWH). While cART has reduced the burden of severe cognitive impairment, mild to moderate cognitive impairment exists in nearly 50% of PWH treated with cART for prolonged periods across all ages [1]. In addition to cognitive sequelae resulting from HIV infection, AIDS, or related treatments, increasing age, coupled with multimorbidity that occurs with aging, creates a risk for declining cognitive function. A study using the Multicenter AIDS Cohort Study (MACS) dataset found that up to 33% of sexual minority men (SMM) with HIV met criteria for mild to moderate cognitive impairment [2]. Another study from MACS that used 5 years of follow-up data found that, while there was no difference in cognitive performance between SMM with or without HIV [3], there was an interaction effect of age and HIV status, such that middle-aged to older SMM with HIV had a significant decline in psychomotor speed and executive function compared with younger SMM with HIV [4]. These findings suggest a need for focused attention on cognitive performance among middle-aged (45–65 years) SMM, as well as older (> 65 years) SMM with and without HIV.

Rather than solely focusing on risk factors, it is important to identify potential protective factors for cognitive function because cognition affects quality of life and everyday functioning [5]. Studies have consistently shown that close relational ties, including romantic, familial, and platonic relationships, affect cognitive function in late life [6–10]. In addition to the presence of such relationships, the *quality* of such relationships is an important contributor to health [11, 12]. The general gerontological literature demonstrates that relationship quality is linked to cognitive function over time and that negative relationship quality may be detrimental to health [13–15]. In romantic partnerships, a review on marital status and cognitive decline suggested that both support and strain from relationships can affect physical health through psychological, biological, and behavioral pathways [16] that are known to contribute to cognitive performance over time. Similar associations have been established in both familial and platonic relationships [17].

With increased societal acceptance of same-sex partnerships and legal changes surrounding marriage equality, there may be changes to relationship structures among SMM that may provide different sources of social support that can be beneficial to cognitive health. Previous research on the social network structures of SMM found that middle-aged and older SMM with or without HIV tend to be single and live alone [18, 19]. A more recent study deriving from the MACS characterized typologies of partnerships among midlife SMM [20]. This study defined primary partners as committed romantic partners, whereas secondary partners were defined as other support members including friends, former/current romantic or sexual partners, and/or biological/chosen family, of whom may provide tangible support such as the provision of financial and material assistance [20]. Forty-nine percent of participants in the study reported having a primary partner and at least 22% reported having secondary partners to rely on for tangible support [20]. Another study using the MACS found a positive association between increased social support and cognitive performance [21]. However, this study assessed the totality of social support derived across *all* relationships within one’s social network (romantic, familial, platonic, and acquaintances) and did not focus on specific relationship types that one could receive perceived and tangible support from. There is a paucity of evidence on how the type and quality of partnerships among midlife SMM are related to cognitive performance. Therefore, we sought to address whether partnership status (having primary and/or secondary partners or both) and partnership quality (support or strain) are associated with cognitive function among midlife and older adult SMM. We hypothesized that having primary or secondary partners or both would be associated with better cognitive performance and that those with no partners would have worse cognitive performance. In addition, we hypothesized that having supportive primary and/or secondary partners would be linked to better cognitive function.

## Methods

### Study Population and Analytic Sample

This was a secondary analysis of a substudy of the MACS known as the understanding the patterns of healthy aging among men who have sex with men substudy. The study designs of the MACS [22, 23] and the substudy [24] have been described elsewhere. The purpose of the MACS was to understand the history of HIV among SMM in the study sites throughout the United States. The MACS began in 1984 and participants attended study visits every 6 months. The substudy sought to understand the psychosocial factors that contribute to healthy aging in middle-aged and older SMM with and without HIV. It included 1318 MACS participants across 6 semiannual visits from 2016 to 2019. Eligibility criteria for the substudy were (1) being at least 40 years of age as of April 2016, (2) completing a MACS visit in the 2 years prior to April 2016, and (3) reporting at least 1 sexual encounter with another man since enrolling in the MACS. The analytic sample for our secondary analysis included 1067 participants living with and without HIV who provided partnership status, partner support/strain, and cognitive performance data across visits 67 (April 2017–September 2017), 68 (October 2017–March 2018), 69 (April 2018–September 2018), and 70 (October 2018–March 2019), contributing a total of 2751 participant-visits.

### Measures

#### Cognitive Performance

The standard Trail Making Test (TMT) is a 2-part (A and B) test used to assess cognitive performance including, but not limited to, attention, visual search, sequencing and shifting, psychomotor speed, and maintenance of 2 simultaneous trains of thought [25]. TMT-A and TMT-B are to be performed as quickly and accurately as possible. In TMT-A, participants draw connecting lines in sequential order between randomly distributed circles numbered from 1 to 25 on a sheet of paper, providing baseline measures in the areas of psychomotor speed, visual search, and target-directed motor tracking. In TMT-B, participants must connect numbers (1–13) and letters (A–M) while alternating between them (i.e., 1-A–2-B–3-C, etc.), testing areas of psychomotor speed, memory, and set-shifting. The test administrator immediately stops the participant when a mistake is made and provides correction. The time to complete TMT-A and TMT-B, measured in seconds, is recorded as a raw score. The Symbol Digit Modalities Test (SDMT) measures information processing speed [25]. Participants use a coded key to match 9 abstract symbols paired with numerical digits. The participant completes the first 10 items with guidance. Then, the participant (without assistance) is assessed to determine how many matched pairings can be made in 90 s. We used *z*-scores (adjusted for age, race/ethnicity, education and second, third and later administration of test) for TMT-A, TMT-B and SDMT, where the *z*-scores represented the number of standard deviations an individual data point is away from the mean. The scores were derived using normative data from participants living without HIV [26]. Positive *z*-scores indicated better cognitive performance (higher than the mean) and negative scores were the opposite (lower than the mean). We summed the TMT-A, TMT-B and SDMT *z*-scores to create an overall measure of cognitive performance, referred to as the *Z*-score composite. Higher scores indicated better overall cognitive performance. Cognitive measures were assessed at visits 67, 68, 69, and 70.

#### Partnership Status

*Partnership status* was derived from self-reported primary and secondary partner status. Primary partner status was assessed from the following question: “Are you currently in a relationship with a primary partner? By primary partner we mean someone who you are committed to above anyone else and with whom you might or might not be having sex.” The answer choices were “yes” or “no.” Participants who answered “yes” were categorized as having a primary partner.

Secondary partner status was obtained from the following question: “We know that some gay and bisexual men form partnerships with other people that can be as intimate or supportive as a primary partnership, but that don’t necessarily include romance or sex. Similar to a primary partner or spouse, this individual might be someone who shares financial resources to pay living expenses, shares housing, shares personal sacred histories between both of you, or takes care of you when seriously ill (or you them). Do you have someone like that in your life currently?” The answer choices were “yes” or “no.” Participants who answered “yes” were categorized as having a secondary partner.

We followed the characterization and distinction of partnerships in the study by Statz et al. [20] to operationalize the types of partnerships, where primary partnerships consisted of commitments typically in a romantic manner and secondary partnerships included close companionships or supportive relationships beyond the primary partnership. As such, partnership status was categorized as follows: (1) no primary or secondary partners; (2) reported secondary partner but no primary partner; (3) reported primary partner but no secondary partner; and (4) reported both primary and secondary partners. Partnership status was assessed at visits 67, 68, 69, and 70.

#### Partnership Quality

*Partnership quality* was measured using a scale adapted from the Midlife in the United States survey [27]. The support and strain subscales were each composed of 4 items and adapted so that they read, “Thinking about your primary (or secondary) partner.”

Partnership support (positive quality) subscale items were as follows: “How much does your partner understand the way you feel about things?”; “How much does your partner really care about you?”; “How much can you rely on your partner for help if you have a serious problem?”; and “How much can you open up to your partner if you need to talk about your worries?”.

Partnership strain (negative quality) subscale items were as follows: “How often does your partner criticize you?”; “How often does your partner make too many demands on you?”; “How often does your partner let you down when you are counting on him/her?”; and “How often does your partner get on your nerves?”.

The responses were on a 5-point Likert scale, ranging from (1) “never” to (5) “regularly.” Scores for each subscale were summed separately and ranged from 4 to 20. Higher values indicate higher partnership support or strain. Participants who reported secondary partnerships were asked the same questions about their secondary partners. Support and strain were assessed at visits 67, 68, 69, and 70.

***Covariates*** were selected based on the previous literature suggesting their associations with cognitive performance. Sociodemographic characteristics were time stable and included the following: age (years, calculated from date of birth and visit date); race and ethnicity (non-Hispanic White /non-Hispanic Black/Hispanic/Other race); and education (less than high school education/high school diploma/at least some college/at least some graduate school). Time-varying self-reported substance use included smoking status (currently smokes/formerly smoked/never smoked), alcohol consumption (binge/moderate to heavy/low to moderate/none), and stimulant use (yes/no). Presence or absence of the following comorbidities were also time-varying and included high blood pressure, depressive symptoms (Center for Epidemiological Studies Depression score ≥ 16), diabetes, dyslipidemia, and kidney disease [28]. HIV serostatus (positive/negative) was derived from enzyme-linked immunosorbent assay with confirmatory Western blot for all MACS participants at their initial visit and at every visit for participants who were HIV negative during the previous visit. Among PWH, we assessed CD4 cell count (cells/mm^3^) and HIV viral load detection. CD4 cell count was categorized into 200 cells/mL^3^ or more and less than 200 cells/mL^3^. Detectable viral load was defined as having plasma HIV RNA levels of greater than 20 copies/mL.

### Statistical Analysis

We generated descriptive statistics on the cognitive outcomes, partnership status and quality, and covariates to overall participants and by HIV status, using frequencies/percentages and medians/IQRs where appropriate. We generated linear mixed models with repeated measures, adjusting for within-participant variance, using SAS procedure PROC MIXED. We examined the relationship between each of the outcomes (first the *Z*-score composite and then individually for TMT-A, TMT-B, SDMT if the composite was significant) and each of the primary predictors (partnership status, primary partner support/strain, and secondary partner support/strain) in separate models. The predictors, outcomes, and covariates were analyzed at each of their respective time points. We first generated bivariate models to test the relationship of the primary predictors and covariates with each cognitive outcome. Additionally, we tested the interaction effect between partnership status and HIV status for each outcome. The final model included the primary predictors, HIV status, race and ethnicity, age, education, and other covariates that had a *p* < 0.10 from the bivariate analysis. Parameter estimates and their respective 95% CI were reported. Of note, the predictors, outcomes, and covariates were analyzed at each time point (visits 67, 68, 69, and 70) as a repeated measures model for a total follow-up of 2 years. All analyses were performed in SAS version 9.4 (Statistical Analysis Software; Cary, NC) with an alpha = 0.05 unless noted otherwise.

#### Missing Data

Of the 2751 participant-visits, 11.3% were missing partnership status, 1.2% were missing TMT-A scores, 1.5% were missing TMT-B scores, and 1.6% were missing SDMT scores. Among the 460 participants reporting at least a primary partner, only 1 (< 0.1%) was missing primary partner support and strain. Among the 161 participants reporting at least a secondary partner, 5 (0.9%) were missing secondary partner support and 6 (1%) were missing secondary partnership strain. These participants were dropped from subsequent respective models. The final sample for assessing the association between partnership status and the 4 outcomes (*Z*-score composite, TMT-A, TMT-B, and SMDT) was 1614 person-visits (*n* = 894 unique men), whereas the models assessing partner support and strain as predictors were limited among the 851 person-visits (*n* = 460 men) who reported a primary partner and the 406 person-visits (*n* = 161 men) who reported a secondary partner, respectively.

## Results

The overall median age at visit 67 was 60.0 years (IQR 54.0–66.0) (Table 1). Overall, 66.9% of participants were non-Hispanic White, 21.7% were non-Hispanic Black, 9.2% were Hispanic, and 2.2% identified as some other race or ethnic category. Most participants had at least some college education or higher (85.7%). Nearly half (49.3%) had previously smoked, 57.7% reported drinking at a low to moderate level, and 9.1% used stimulants. The presences of comorbidities were as follows: hypertension (56.3%), depressive symptoms (22.0%), diabetes (13.3%), dyslipidemia (75.0%), and kidney disease (17.3%). Among PWH, 2.4% had CD4 cell count less than 200 cells/mL^3^ and 10.6% had a detectable viral load.

**Table 1:** Sample characteristics by HIV status

	HIV negative (*n* = 521)	HIV positive (*n* = 546)	All (*N* = 1067)
Age at visit 67, median (IQR) in years	63.0 (57.0–69.0)	58.0 (53.0–64.0)	60.0 (54.0–66.0)
*Race and ethnicity, n (%)*			
Non-Hispanic white	416 (79.8)	298 (54.6)	714 (66.9)
Non-Hispanic black	70 (13.4)	162 (29.7)	232 (21.7)
Hispanic	24 (4.6)	74 (13.6)	98 (9.2)
Other	11 (2.1)	12 (2.2)	23 (2.2)
*Education, n (%)*			
Less than high school education	9 (1.7)	30 (5.5)	39 (3.7)
High school education	38 (7.3)	76 (13.9)	114 (10.7)
At least some college	249 (47.8)	292 (53.5)	541 (50.7)
At least some graduate school	225 (43.2)	148 (27.1)	373 (35.0)
*Smoke status, n (%)*^*a*^			
Currently smokes	135 (9.9)	297 (21.4)	432 (15.7)
Formerly smoked	720 (52.7)	636 (45.9)	1356 (49.3)
Never smoked	494 (36.2)	425 (30.7)	919 (33.4)
Missing	16 (1.2)	28 (2.0)	44 (1.6)
*Drink category, n (%)*^*a*^			
Binge	42 (3.1)	67 (4.8)	109 (4.0)
Low/moderate	851 (62.3)	736 (53.1)	1587 (57.7)
Moderate/heavy	184 (13.5)	208 (15.0)	392 (14.2)
None	257 (18.8)	326 (23.5)	583 (21.2)
Missing	31 (2.3)	49 (3.5)	80 (2.9)
*Stimulant use, n (%)*^*a*^			
No	1284 (94.1)	1142 (82.4)	2426 (88.2)
Yes	53 (3.9)	197 (14.2)	250 (9.1)
Missing	28 (2.1)	47 (3.4)	75 (2.7)
*High blood pressure, n (%)*^*a*^			
No	567 (41.5)	587 (42.4)	1154 (41.9)
Yes	783 (57.4)	767 (55.3)	1550 (56.3)
Missing	15 (1.1)	32 (2.3)	47 (1.7)
*Depressive symptom, n (%)*^*a*^			
No	1097 (80.4)	981 (70.8)	2078 (75.5)
Yes	230 (16.8)	374 (27.0)	604 (22.0)
Missing	38 (2.8)	31 (2.2)	69 (2.5)
*Diabetes, n (%)*^*a*^			
No	1169 (85.6)	1148 (82.8)	2317 (84.2)
Yes	163 (11.9)	204 (14.7)	367 (13.3)
Missing	33 (2.4)	34 (2.5)	67 (2.4)
*Dyslipidemia, n (%)*^*a*^			
No	288 (21.1)	285 (20.6)	573 (20.8)
Yes	1037 (76.0)	1025 (74.0)	2062 (75.0)
Missing	40 (2.9)	76 (5.5)	116 (4.2)
*Kidney disease, n (%)*^*a*^			
No	1210 (88.6)	1012 (73.0)	2222 (80.8)
Yes	133 (9.7)	342 (24.7)	475 (17.3)
Missing	22 (1.6)	32 (2.3)	54 (2.0)
*CD4 cell count, n (%)*^*a*^			
≥ 200 cells/mL^3^	–	1335 (96.3)	1335 (48.5)
< 200 cells/mL^3^	–	33 (2.4)	33 (1.2)
Missing	1365 (100.0)	18 (1.3)	1383 (50.3)
*Viral load detection, n (%)*^*a*^			
No	–	1072 (77.3)	1073 (39.0)
Yes	–	291 (21.0)	291 (10.6)
Missing	1365 (100)	23 (1.7)	1387 (50.4)
Trail making test part A, in seconds, median (IQR)^a^	20.0 (16.0–25.0)	21.0 (17.0–26.0)	20.0 (16.0–26.0)
Trail making test part B, in seconds, median (IQR)^a^	42.0 (32.0–57.0)	46.0 (34.0–64.0)	44.0 (33.0–60.0)
Symbol digit modalities test, raw score, median (IQR)^a^	53.0 (45.0–63.0)	51.0 (42.0–61.0)	52.0 (44.0–62.0)
Trail making test part A, *z*-score, median (IQR)^a, b^	0.09 (− 0.63 to 0.86)	0.20 (− 0.66 to 0.90)	0.15 (− 0.64 to 0.87)
Trail making test part B, *z*-score, median (IQR)^a, b^	0.14 (− 0.66 to 0.90)	0.08 (− 0.77 to 0.76)	0.11 (− 0.70 to 0.90)
Symbol digit modalities test, *z*-score, median (IQR)^a, b^	− 0.01 (− 0.76 to 0.72)	− 0.01 (− 0.75 to 0.76)	− 0.01 (− 0.75 to 0.73)
*Z*-score composite (TMT-A, B and SDMT), median (IQR)^a, b^	0.28 (− 1.44 to 1.84)	0.23 (− 1.62 to 1.98)	0.25 (− 1.56 to 1.94)
*Primary partnership, n (%)*^*a*^			
Yes	719 (52.7)	551 (39.8)	1270 (46.2)
No	578 (42.3)	742 (53.5)	1320 (48.0)
Missing	68 (5.0)	93 (6.7)	161 (5.9)
*Secondary partnership, n (%)*^*a*^			
Yes	292 (21.4)	301 (21.7)	593 (21.6)
No	943 (69.1)	914 (66.0)	1857 (67.5)
Missing	130 (9.5)	171 (12.3)	301 (10.9)
*Partnership status, n (%)*^*a*^			
No partnership	411 (30.1)	529 (38.2)	940 (34.2)
Primary only	529 (38.8)	379 (27.3)	908 (33.0)
Secondary only	138 (10.1)	161 (11.6)	299 (10.9)
Both primary and secondary	154 (11.3)	138 (10.0)	292 (10.6)
Missing	133 (9.7)	179 (12.9)	312 (11.3)
Primary partner support, median (IQR)^a^	16.0 (15.0–19.0)	16.0 (14.0–18.0)	16.0 (15.0–19.0)
Primary partner strain, median (IQR)^a^	8.0 (7.0–10.0)	9.0 (7.0–11.0)	9.0 (7.0–11.0)
Secondary partner support, median (IQR)^a^	16.0 (14.0–17.0)	16.0 (14.0–16.0)	16.0 (14.0–17.0)
Secondary partner strain, median (IQR)^a^	8.0 (6.0–10.0)	8.0 (6.0–10.0)	8.0 (6.0–10.0)

^a^Participant visits: 1365 among HIV-negative participants and 1386 among HIV-positive participants

^b^*Z*-scores for TMT-A, -B, and SDMT were demographically adjusted by age, race, education and later administration of the testing prior to calculating composite score

Overall, 46.2% reported a primary partner and 21.6% reported a secondary partner. Partnership status categories were as follows: 34.2% had no partnerships, 33.0% had only a primary partner, 10.9% had only a secondary partner, and 10.6% had both primary and secondary partners. The median primary partner support and strain scores were 16.0 (IQR: 15.0–19.0) and 9.0 (IQR: 7.0–11.0), respectively. The median secondary partner support and strain scores were 16.0 (IQR: 14.0–17.0) and 8.0 (IQR: 6.0–10.0), respectively. The median overall *Z*-score composite was 0.25 (− 1.56 to 1.94). The median adjusted *z*-scores for TMT-A, TMT-B, and SDMT were 0.15 (IQR − 0.64 to 0.87), 0.11 (IQR − 0.70 to 0.90) and -0.01 (− 0.75 to 0.73), respectively. The median raw scores for TMT-A and TMT-B were 20.0 (IQR, 16.0–26.0) and 44.0 (IQR, 33.0–60.0), respectively. The median raw SDMT score was 52.0 (IQR, 44.0–62.0). Details by HIV status are reported in Table 1.

In unadjusted models (Table 2), reporting a primary partner was associated with better cognitive performance as demonstrated in each of the cognitive outcome: (1) *Z*-score composite ($\widehat{\upbeta }$= 0.76 [95% CI 0.52–1.01]); (2) TMT-A ($\widehat{\upbeta }$= 0.29 [95% CI 0.18–0.40]); (3) TMT-B ($\widehat{\upbeta }$= 0.27 [95% CI 0.16–0.38]); and (4) SDMT ($\widehat{\upbeta }$= 0.23 [95% CI 0.11–0.35]). An increase in the primary partner support score was positively associated only with TMT-A ($\widehat{\upbeta }$= 0.02 [95% CI 0.00–0.05]). An increase in secondary partner support score was positively associated with the *Z*-score composite ($\widehat{\upbeta }$= 0.13 [95% CI 0.05–0.22]), TMT-A ($\widehat{\upbeta }$= 0.05 [95% CI 0.01–0.09]), and TMT-B ($\widehat{\upbeta }$= 0.05 [95% CI 0.01–0.09]). Covariates associated with better cognitive performance (positive parameter values) included Hispanic or Other racial/ethnic background, high school or some college education, drinking status, and dyslipidemia. Covariates associated with worsen cognitive performance (negative parameter values) included increasing age, being current smoker, using stimulants, as well as select comorbid conditions (HCV-positive, hypertension, diabetes and kidney disease). Details of their association are reported in Table 2.

**Table 2:** Bivariate associations of partnership status (*N* = 894), primary partner support/strain (*N* = 460), secondary partner support/strain (*N* = 161) on cognitive function over visits 67, 68, 69, and 70 among healthy aging substudy participants with nonmissing data

	TMT-A^a^ $\widehat{\beta }$ (95% CI)	TMT-B^a^ $\widehat{\beta }$ (95% CI)	SDMT^a^ $\widehat{\beta }$ (95% CI)	*Z*-Score Composite^a^ $\widehat{\beta }$ (95% CI)
*Partnership status*				
Both primary and secondary	0.07 (95% CI − 0.09 to 0.23)	0.06 (95% CI − 0.10 to 0.22)	0.13 (95% CI − 0.04 to 0.30)	0.25 (95% CI − 0.11 to 0.60)
Primary only	**0.29 (95% CI 0.18–0.40)**	**0.27 (95% CI 0.16–0.38)**	**0.23 (95% CI 0.11–0.35)**	**0.76 (95% CI 0.52–1.01)**
Secondary only	0.01 (95% CI − 0.15 to 0.17)	− 0.05 (95% CI − 0.21 to 0.11)	− 0.06 (95% CI − 0.24 to 0.11)	− 0.10 (95% CI − 0.45 to 0.25)
No partnership (referent)				
*Primary partner support and strain*				
Primary partner support	**0.02 (95% CI 0.00–0.05)**	0.02 (95% CI 0.00–0.05)	0.00 (95% CI − 0.03 to 0.02)	0.04 (95% CI − 0.01 to 0.09)
Primary partner strain	0.01 (95% CI − 0.02 to 0.03)	0.02 (95% CI − 0.01 to 0.04)	− 0.01 (95% CI − 0.03 to 0.02)	0.02 (95% CI − 0.04 to 0.08)
*Secondary partner support and strain*				
Secondary partner support	**0.05 (95% CI 0.01–0.09)**	**0.05 (95% CI 0.01–0.09)**	0.04 (95% CI 0.00–0.08)	**0.13 (95% CI 0.05–0.22)**
Secondary partner strain	0.02 (95% CI − 0.01 to 0.06)	0.02 (95% CI − 0.01 to 0.06)	0.00 (95% CI − 0.04 to 0.04)	0.05 (95% CI − 0.04 to 0.13)
*HIV status*				
Positive	0.02 (95% CI − 0.07 to 0.11)	− 0.08 (95% CI − 0.17 to 0.01)	− 0.02 (95% CI − 0.12 to 0.08)	− 0.08 (95% CI − 0.28—0.13)
Negative (Referent)				
Age at Visit (per 1-year increase)	**− 0.03 (95% CI** − **0.03 to** − **0.02)**	**− 0.03 (95% CI** − **0.04 to** − **0.02)**	**− 0.02 (95% CI − 0.02 to − 0.01)**	**− 0.07 (95% CI − 0.08 to − 0.06)**
*Race ethnicity*				
Black, non-Hispanic	− 0.09 (95% CI − 0.20 to 0.02)	− 0.03 (95% CI − 0.15—0.08)	**− 0.16 (95% CI − 0.28 to0.04)**	**− 0.27 (95% CI − 0.52 to − 0.02)**
Hispanic	**0.29 (95% CI 0.13–0.45)**	− 0.16 (95% CI − 0.32 to 0.00)	− 0.10 (95% CI − 0.28 to 0.07)	0.01 (95% CI − 0.35 to 0.38)
Other race	**0.69 (95% CI 0.39–1.00)**	0.03 (95% CI − 0.28 to 0.34)	0.26 (95% CI − 0.07 to 0.59)	**0.94 (95% CI 0.24–1.64)**
White, non-Hispanic (Referent)				
*Education*				
At least some college	**0.13 (95% CI 0.03–0.23)**	**0.17 (95% CI 0.07–0.27)**	0.03 (95% CI − 0.08 to 0.14)	**0.31 (95% CI 0.09–0.54)**
HS Education	**0.28 (95% CI 0.12–0.43)**	0.15 (95% CI − 0.01 to 0.31)	− 0.16 (95% CI − 0.32 to 0.01)	0.26 (95% CI − 0.10 to 0.61)
Less than HS education	0.22 (95% CI − 0.01 to 0.46)	− 0.01 (95% CI − 0.25 to 0.23)	− 0.04 (95% CI − 0.29—0.22)	0.17 (95% CI − 0.37 to 0.71)
At least some graduate school (Referent)				
*Smoke status*				
Currently smokes	**− 0.17 (95% CI − 0.31 to − 0.03)**	**− 0.25 (95% CI − 0.39 to − 0.11)**	**− 0.25 (95% CI − 0.40 to − 0.10)**	**− 0.66 (95% CI − 0.97 to − 0.35)**
Formerly smoked	0.01 (95% CI − 0.10 to 0.11)	− 0.01 (95% CI − 0.12 to 0.09)	− 0.11 (95% CI − 0.21 to 0.00)	− 0.11 (95% CI − 0.34 to 0.12)
Never smoked (Referent)				
*Drink category*				
Binge	**0.31 (95% CI 0.06–0.55)**	0.01 (95% CI − 0.25 to 0.26)	**0.45 (95% CI 0.19–0.72)**	**0.75 (95% CI 0.19–1.31)**
Low/Moderate	0.11 (95% CI − 0.01 to 0.22)	0.04 (95% CI − 0.08 to 0.15)	**0.18 (95% CI 0.06–0.31)**	**0.31 (95% CI 0.05–0.57)**
Moderate/Heavy	**0.33 (95% CI 0.18–0.49)**	0.16 (95% CI 0.00–0.32)	**0.36 (95% CI 0.20—0.53)**	**0.83 (95% CI 0.48—1.18)**
None (Referent)				
*Stimulant use*				
Yes	− 0.14 (95% CI − 0.30 to 0.01)	**− 0.17 (95% CI − 0.33 to − 0.01)**	**− 0.24 (95% CI − 0.41–0.07)**	**− 0.53 (95% CI − 0.89 to − 0.17)**
No (Referent)				
*HCV*				
Positive	**− 0.44 (95% CI − 0.68 to − 0.20)**	**− 0.63 (95% CI − 0.87 to − 0.38)**	**− 0.37 (95% CI − 0.63 to − 0.12)**	**− 1.39 (95% CI − 1.92 to − 0.85)**
Negative (Referent)				
*High blood pressure*				
Yes	− 0.08 (95% CI − 0.17 to 0.01)	**− 0.13 (95% CI − 0.23 to − 0.04)**	− 0.06 (95% CI − 0.16 to 0.04)	**− 0.27 (95% CI − 0.48 to − 0.06)**
No (Referent)				
Diabetes				
Yes	**− 0.38 (95% CI − 0.52 to − 0.25)**	**− 0.35 (95% CI − 0.48 to − 0.21)**	**− 0.31 (95% CI − 0.45 to − 0.16)**	**− 1.00 (95% CI − 1.31 to − 0.70)**
No (Referent)				
*Dyslipidemia*				
Yes	0.08 (95% CI − 0.03 to 0.20)	0.10 (95% CI − 0.02 to 0.21)	**0.14 (95% CI 0.02–0.26)**	**0.31 (95% CI 0.05–0.56)**
No (Referent)				
*Kidney disease*				
Yes	**− 0.36 (95% CI − 0.48 to − 0.24)**	**− 0.37 (95% CI − 0.49 to − 0.25)**	**− 0.34 (95% CI − 0.47–0.21)**	**− 1.03 (95% CI − 1.30 to − 0.76)**
No (Referent)				

Bold indicates the statistically significant estimates using the 95% confidence interval (*p* <.05)

^a^*Z*-scores for TMT-A, -B, and SDMT were demographically adjusted by age, race, education and later administration of the testing prior to calculating composite score

**Fig. 1:**
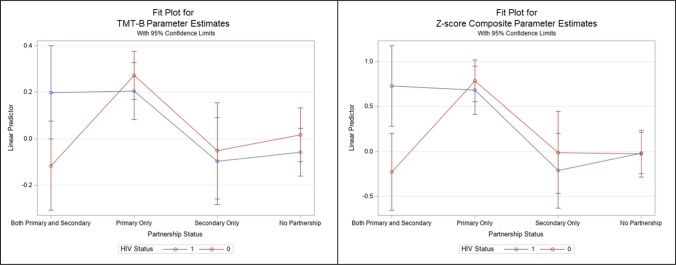
Interaction plot of partnership status and HIV status on TMT-B and *Z*-score composite

The interaction effect between partnership status and HIV status was statistically significant for the* Z*-score composite ($\widehat{\upbeta }$= 0.94 [95% CI 0.23–1.65]) and TMT-B ($\widehat{\upbeta }$= 0.39 [95% CI 0.07–0.71]) (Table 3; Fig. 1). We also observed a similar association with the SDMT outcome ($\widehat{\upbeta }$= 0.35 [95% CI 0.00–0.69]), albeit only marginally significant.

**Table 3:** Interaction of partnership status and HIV status on cognitive function over visits 67, 68, 69, and 70 among healthy aging substudy participants with nonmissing data

	TMT-A^a^ $\widehat{\beta }$ (95% CI)	TMT-B^a^ $\widehat{\beta }$ (95% CI)	SDMT^a^ $\widehat{\beta }$ (95% CI)	*Z*-Score Composite^a^ $\widehat{\beta }$ (95% CI)
*Partnership status*				
Both primary and secondary	− 0.04 (95% CI − 0.27 to 0.19)	− 0.13 (95% CI − 0.36 to 0.09)	− 0.03 (95% CI − 0.28 to 0.21)	− 0.20 (95% CI − 0.70 to 0.30)
Primary only	**0.33 (95% CI 0.17–0.49)**	**0.26 (95% CI 0.10–0.41)**	**0.26 (95% CI 0.09–0.43)**	**0.81 (95% CI 0.46–1.16)**
Secondary only	0.13 (95% CI − 0.11 to 0.37)	− 0.07 (95% CI − 0.31 to 0.17)	− 0.03 (95% CI − 0.29 to 0.22)	0.01 (95% CI − 0.51 to 0.54)
No partnership (referent)				
*HIV status*				
Positive	0.08 (95% CI − 0.08 to 0.24)	− 0.07 (95% CI − 0.23 to 0.08)	0.01 (95% CI − 0.16 to 0.18)	0.01 (95% CI − 0.34 to 0.36)
Negative (referent)				
*Partnership status X HIV status*				
Both primary and secondary*positive	0.23 (95% CI − 0.09 to 0.55)	**0.39 (95% CI 0.07–0.71)**	0.35 (95% CI 0.00–0.69)	**0.94 (95% CI 0.23–1.65)**
Both primary and secondary*negative (referent)	–	–	–	
Primary only*positive	− 0.06 (95% CI − 0.29 to 0.16)	0.01 (95% CI − 0.22 to 0.23)	− 0.06 (95% CI − 0.31 to 0.18)	− 0.11 (95% CI − 0.61—0.39)
Primary only*negative (referent)	–	–	–	
Secondary only*positive	− 0.21 (95% CI − 0.53 to 0.11)	0.03 (95% CI − 0.29 to 0.35)	− 0.06 (95% CI − 0.40 to 0.29)	− 0.21 (95% CI − 0.92 to 0.50)
Secondary only*negative (referent)	–	–	–	

Bold indicates the statistically significant estimates using the 95% confidence interval (*p* <.05)

*SDMT* Symbol digit modalities test; *TMT* trail making test

^a^*Z*-scores for TMT-A, -B, and SDMT were demographically adjusted by age, race, education and later administration of the testing prior to calculating composite score

In adjusted models, having a primary partner was associated with better cognitive performance as demonstrated in the *Z*-score composite ($\widehat{\upbeta }$= 0.41 [95% CI 0.12–0.70]), TMT-A ($\widehat{\upbeta }$= 0.16 [95% CI 0.02–0.30]), and TMT-B ($\widehat{\upbeta }$= 0.19 [95% CI 0.06–0.33]). Secondary partner support was also associated with better cognitive performance shown in the *Z*-score composite ($\widehat{\upbeta }$= 0.13 [95% CI 0.03–0.23]) and TMT-A ($\widehat{\upbeta }$= 0.06 [95% CI 0.01–0.10]) models (Table 4).

**Table 4:** Adjusted associations of partnership status (*N* = 894), primary partner support/strain (*N* = 460), secondary partner support/strain (*N* = 161) on cognitive function over visits 67, 68, 69, and 70 among healthy aging substudy participants with nonmissing data

	TMT-A^b^ $\widehat{\beta }$ (95% CI)	TMT-B^b^ $\widehat{\beta }$ (95% CI)	SDMT^b^ $\widehat{\beta }$ (95% CI)	*Z*-Score Composite^a^ $\widehat{\beta }$ (95% CI)
*Partnership status*				
Both primary and secondary	− 0.04 (95% CI − 0.23 to 0.15)	− 0.02 (95% CI − 0.20 to 0.16)	0.01 (95% CI − 0.20 to 0.22)	− 0.10 (95% CI − 0.50 to 0.30)
Primary only	**0.16 (95% CI 0.02–0.30)**	**0.19 (95% CI 0.06–0.33)**	0.11 (95% CI − 0.04 to 0.26)	**0.41 (95% CI 0.12–0.70)**
Secondary only	− 0.02 (95% CI − 0.20 to 0.17)	0.00 (95% CI − 0.18 to 0.18)	− 0.04 (95% CI − 0.24 to 0.17)	− 0.09 (95% CI − 0.48 to 0.31)
No partnership (referent)				
*Primary partner support and strain*				
Primary partner support	0.00 (95% CI − 0.03 to 0.03)	0.01 (95% CI − 0.02 to 0.03)	− 0.02 (95% CI − 0.06 to 0.01)	− 0.02 (95% CI − 0.08 to 0.04)
Primary partner strain	− 0.01 (95% CI − 0.04 to 0.02)	0.02 (95% CI − 0.01 to 0.05)	− 0.01 (95% CI − 0.05 to 0.03)	0.00 (95% CI − 0.06 to 0.07)
*Secondary partner support and strain*				
Secondary partner support	**0.06 (95% CI 0.01–0.10)**	0.05 (95% CI 0.00–0.09)	0.05 (95% CI 0.00–0.09)	**0.13 (95% CI 0.03–0.23)**
Secondary partner strain	0.04 (95% CI − 0.01 to 0.09)	0.05 (95% CI 0.00–0.09)	0.02 (95% CI − 0.02 to 0.07)	0.09 (95% CI 0.00–0.19)

Bold indicates the statistically significant estimates using the 95% confidence interval (*p* <.05)

*SDMT* Symbol digit modalities test; *TMT* trail making test

Adjusted for by HIV status, age, race and ethnicity, education, drink category, smoking status, stimulant use, depressive symptoms, hepatitis C status, high blood pressure, diabetes, dyslipidemia, and kidney disease

^b^*Z*-scores for TMT-A, -B, and SDMT were demographically adjusted by age, race, education and later administration of the testing prior to calculating composite score

## Discussion

This study aimed to estimate the associations between the partnership type and quality on cognitive performance among midlife SMM with and without HIV. Our adjusted model showed that primary partnership was associated with greater cognitive performance as indicated by the *Z*-score composite. When individual tests were examined, TMT-A and TMT-B were significant, indicating that primary partnership was related to better attention, visual search, and psychomotor speed. These results suggest the buffering effects of primary partnership status on cognitive performance for midlife SMM. When considering the quality of the partnerships, only the secondary partner's supportive quality was related to better cognitive performance. Additionally, there was an interaction effect between partnership status and HIV status on the *Z*-score composite and TMT-B such that among middle-aged and older SMM with HIV, those with both primary and secondary partnerships had better performances on these cognitive outcomes than those without any partners. This underscores the potential protective effects of having multiple supportive partnerships on cognition in this subsample.

Our findings align with extant gerontological literature supporting the positive effects of marital status and quality on cognitive function [6–12, 28–30]. Just as among heterosexual couples, primary partnership among SMM may serve as the key source of social integration, which may require intensive cognitive demands and processing for consistent interactions and efforts to maintain such relationships, thereby rendering protective effects on cognitive function. This is also consistent with the findings in an overlapping sample of midlife SMM by Henderson et al. [21], where they found increased social support derived from interpersonal relationships (e.g., romantic, familial, and platonic) were positively associated with greater psychomotor and information processing ability over a 2-year period. Additionally, previous research has shown a beneficial effect of relationship status on overall health, which may be reflected in cognitive performance and/or other underlying factors that affect overall cognition [31].

Our findings showed that primary partnership status was associated with TMT-A and B, a measure of attention, visual search, sequencing and shifting, and psychomotor speed, which are cognitive domains that show typical aging-related declines. Aging is associated with declines in speed and information processing ability, and this decline may, in part, cause the classic aging-related cognitive changes [32, 33]. Our findings indicate that having a primary partner was associated with a 0.16 increase in TMT-A *z*-score and a 0.19 increase in TMT-B *z*-score. While a difference of these *z*-scores is not large from a clinical perspective, this is a cohort of generally healthy men in their 50 s and early 60 s. We would not expect to see high rates of overt cognitive impairment at this stage of midlife and, as such, even associations of small magnitude are of interest from a prevention standpoint.

There was a significant interaction effect of partnership status and HIV status on cognition—such that PWH who had both primary and secondary partners had better performance on the *Z*-score composite and TMT-B than PWH who had no partnership. TMT-B is a commonly used measure to assess both psychomotor speed and executive functioning (specifically set-shifting), whereas both SDMT and TMT-A predominantly evaluate psychomotor speed and sustained visual search, though SDMT also includes a more modest working memory component [4]. Therefore, our findings highlight the potential for having multiple partners buffering speeded set-shifting/executive function changes more so than basic psychomotor speed in midlife SMM with HIV.

Secondary partnership status did not have any direct associations on all cognitive function tests in our adjusted models. Because of the way we assessed secondary partnership status, we speculate that while secondary partners may provide tangible, instrumental support, they may not necessarily provide all other aspects of support that are important for preserving cognitive health as much as primary partners do. Primary partners may be the primary resource for not only providing financial, but also informational and emotional support that are needed to age well [34]. We speculate that the comprehensive support that primary partners provide may, in turn, allow for mental and cognitive benefits.

We hypothesized that the partnership quality would be associated with cognitive outcomes. While supportive quality from both primary and secondary partners was related to some cognitive outcomes in bivariate models, the statistical significance tapered off after introducing other covariates in our adjusted models. Only supportive quality of the secondary partners was associated with some aspects of cognitive outcomes (TMT-A and the *Z*-score composite). Prior studies of heterosexual older adults suggest that quality of marital relationship is related to better cognitive performance [11–13, 15], but our findings did not support these results. This could potentially be due, in part, to not enough variability in the quality of partnerships in our sample. For example, our sample generally reported a high level of partner support (median score of 16 of a total possible score of 20 to indicate high support) and low level of partner strain (median score of 8 of a total possible score of 20 to indicate high strain) regardless of partnership types, reflecting that overall, our sample of partnered SMM had supportive relationships with their partners. Additionally, most participants performed within normal, expected ranges on the cognitive measures. It may be the case that among SMM with low cognitive scores or with frank cognitive impairment, any associations of partnership on cognitive performance would be larger. Finally, we did not assess for marital relationships, but centered around partnership relationships, which is contextually and conceptually distinct.

There are limitations to consider when interpreting our findings. First, the MACS and the substudy included SMM residing in the United States. Therefore, the findings may not be generalizable to sexual gender minority women or other men living with HIV and without HIV and those outside of the United States. Second, we did not have a comprehensive cognitive evaluation, limiting our ability to test the effect of partnership on different cognitive domains. While TMT-A, TMT-B, and SDMT are robust measures for certain domains/skill sets of cognition (e.g., psychomotor speed and executive processing), these measures do not assess other domains of cognition such as memory, language, fine motor, or other aspects of executive function. However, we utilized *z*-scores that controlled for demographics and practice effects on cognitive performance. We also formulated a composite score that reflects these three cognitive variables which rely upon both distinct and overlapping cognitive constructs. By first examining a cognitive composite and then decomposing this variable to examine the individual contribution of each measure, we were able to see whether overarching cognitive contributions or underlying constructs were at play. Future studies should investigate whether and how partnership status and quality are associated with different domains of cognitive function by using a comprehensive neurocognitive test battery that allows for more detailed assessment of specific cognitive performance. Third, because the measures we used to define partnership status and quality were created for our study, they have not been standardized and the psychometric properties are thus unknown. We were unable to capture whether individuals who reported not having any current primary partners had partners previously and, if so, whether they experienced loss in the past. The study did not capture the frequency of the contact with partners and what kind of support these partners do or do not provide, further limiting our understanding about the active ingredient influencing the impact of partnership status on cognition among midlife and older SMM. Furthermore, the quality of relationships may change over time with differing situations, but this was not assessed. SMM may have diverse support networks for which we were unable to control, which may have confounded our findings. More research is needed to accurately reflect and assess for diverse types of partnership and the dynamic nature and experiences of partnership and social connections for SMM through the life course [35]. Longitudinal dyadic data would yield comprehensive information about how partnership quality, frequency of interactions, and cognitive function are related and change over time. Finally, it should be noted that most scores on the cognitive tests assessed here were within normal expected ranges and did not necessarily indicate a frank cognitive impairment.

## Conclusions

Our findings add to existing evidence that primary partnership benefits cognitive performance among midlife SMM with or without HIV. We also found a positive association between supportive quality of the secondary partner and cognitive performance. Our results align with the positive effects of supportive and positive marital relationships on cognitive function among general older adults. Having a primary partner serves as a protector to cognitive performance among midlife SMM both with and without HIV. For those with HIV, having both primary and secondary partners were associated with better cognitive outcomes than those with no partners at all. Community programs and counseling services that focus on relationship building, especially on strategies to enhance supportive and positive partnership quality need to be developed and offered from the organizations that assist midlife and older SMM. Further, future studies should identify the protective mechanisms by which partnership status can improve cognition among this growing subpopulation of older adults.
